# Depth and substratum differentiations among coexisting herbivorous cichlids in Lake Tanganyika

**DOI:** 10.1098/rsos.160229

**Published:** 2016-11-16

**Authors:** Hiroki Hata, Haruki Ochi

**Affiliations:** 1 Graduate School of Science and Engineering, Ehime University, 2–5 Bunkyo, Matsuyama, Ehime, Japan; 2 4-4-7 Higashimon-cho, Imabari 794-0033, Japan

**Keywords:** African cichlid, adaptive radiation, habitat depth, substratum inclination, niche differentiation, ecomorph

## Abstract

Cichlid fish in Lake Tanganyika represent a system of adaptive radiation in which eight ancestral lineages have diversified into hundreds of species through adaptation to various niches. However, Tanganyikan cichlids have been thought to be oversaturated, that is, the species number exceeds the number of niches and ecologically equivalent and competitively even species coexist. However, recent studies have shed light on niche segregation on a finer scale among apparently equivalent species. We observed depth and substratum preferences of 15 herbivorous cichlids from four ecomorphs (i.e. grazer, browser, scraper and scooper) on a rocky littoral slope for 14 years. Depth differentiation was detected among grazers that defended feeding territories and among browsers with feeding territories. Cichlid species having no feeding territory also showed specificity on depth and substratum, resulting in habitat segregation among species that belong to the same ecomorph. Phylogenetically close species did not occupy adjacent depths, nor the opposite depth zones. Our findings suggest that apparently equivalent species of the same ecomorph coexist parapatrically along depth on a few-metre scale, or coexist with different substratum preferences on the rocky shore, and this niche segregation may have been acquired by competition between encountering equivalent species through repetitive lake-level fluctuations.

## 1. Introduction

The cichlid species in Lake Tanganyika represent a magnificent system of adaptive radiation, which is the evolution of a number of divergent species from a common ancestor as a consequence of their adaptation to various ecological niches. Since the formation of the lake basin *ca* 9–12 Ma, more than 200 species have diverged from eight colonizing lineages [1–4]. Up to 15 species of herbivorous cichlids coexist on a rocky littoral slope of the lake [5–7]. These cichlids include 10 species of the tribe Tropheini, three Lamprologini, one Ectodini, one Eretmodini (table 1). Therefore, the herbivorous fish community has been established through repetitive adaptations to herbivory by these cichlid tribes [4].

**Table 1.** Herbivorous cichlids in Lake Tanganyika and their ecomorphs, territoriality and breeding habits.

tribe	species	abbreviation	feeding ecomorph	feeding territory	breeding	references
Ectodini	*Xenotilapia papilio*	Xpap	scooper	breeding pairs only	mouth-brooder	[8,9]
Eretmodini	*Eretmodus cyanostictus*	Ecya	scraper	breeding pairs only	mouth-brooder	[10,11]
Lamprologini	*Telmatochromis temporalis*	Ttem	browser	yes	substrate-brooder	[12,13]
Lamprologini	*Telmatochromis vittatus*	Tvit	browser	no	substrate-brooder	[14]
Lamprologini	*Variabilichromis moorii*	Vmoo	browser	yes	substrate-brooder	[15]
Tropheini	*Interochromis loocki*	Iloo	grazer	dominant males only	mouth-brooder	[16]
Tropheini	*Limnotilapia dardennii*	Ldar	browser	no	mouth-brooder	[12]
Tropheini	*Pseudosimochromis curvifrons*	Pcur	browser	dominant males only	mouth-brooder	[12,17]
Tropheini	*Petrochromis famula*	Pfam	grazer	dominant males only	mouth-brooder	[18]
Tropheini	*Petrochromis fasciolatus*	Pfas	grazer	dominant males only	mouth-brooder	[18–20]
Tropheini	*Petrochromis polyodon*	Ppol	grazer	yes	mouth-brooder	[12]
Tropheini	*Petrochromis horii*	Phor	grazer	yes	mouth-brooder	[21]
Tropheini	*Petrochromis trewavasae*	Ptre	grazer	yes	mouth-brooder	[12]
Tropheini	*Simochromis diagramma*	Sdia	browser	no	mouth-brooder	[12]
Tropheini	*Tropheus moorii*	Tmoo	browser	yes	mouth-brooder	[12,22,23]

Tanganyikan cichlids are unique in the richness of convergent forms that evolved in the lake and coexist in similar habitats [4]. In cichlid adaptive radiation, specialists are thought to have evolved from generalist riverine ancestors [24], and specialist species can be grouped into particular ecomorphs, i.e. species with the same structural habitat/niche, similar in morphology and behaviour, but not necessarily close phylogenetic relatedness [25]. Five tribes of the family have acquired four herbivorous ecomorphs: grazers, browsers, scoopers and scrapers [12,26–28]. Grazers comb epiphytic unicellular algae attached to epilithic assemblages using multiple rows of similarly sized slender teeth with fork-like tricuspid tips [29,30]. Browsers nip and nibble filamentous algae using their bicuspid teeth, which line the outermost edges of both jaws [22,31]. Scoopers scoop up portions of sand by protrusion of upper jaw composed of the enlarged maxilla, then sort and discard the coarser particles through their gill covers and ingest algal material [32,33]. Their teeth are small and unicuspid, and outer teeth are arranged in a single regular row in the outmost edges of both jaws, and inner teeth are smaller and arranged in a few regular or irregular rows [13,34]. Scrapers rub epiphyton from rock surfaces using several rows of chisel-like teeth [35]. The fishes of each ecomorph have distinct specialized morphologies, such as body shape [36], jaw structure [26,37,38] and intestine length [38–40], physiological abilities, such as secretion of specific digestive enzymes [39], and behaviours, such as cropping frequency, substratum choice [12,22] and territoriality [17,18].

Sympatric herbivorous species compete with each other for feeding space, and direct competition is more frequent and severe among species of the same ecomorph [12,27,41]. In this situation, how do similar species segregate their niches and coexist? Niche segregation leading to multi-species coexistence has at least three axes: space, food and time [42]. These herbivorous cichlids do not separate active times during the day or seasonally [43]. Here we focus on space, such as water depth and substratum type. Depth is an important axis of niche differentiation, and depth segregation can lead to speciation in fishes in both marine and lacustrine environments [44,45]. It is well known that depth-specific divergence in male mating colours and female colour preferences, as well as depth-specific ecological adaptations, can be key factors in cichlid speciation in Lake Victoria [46].

**Figure 1.**
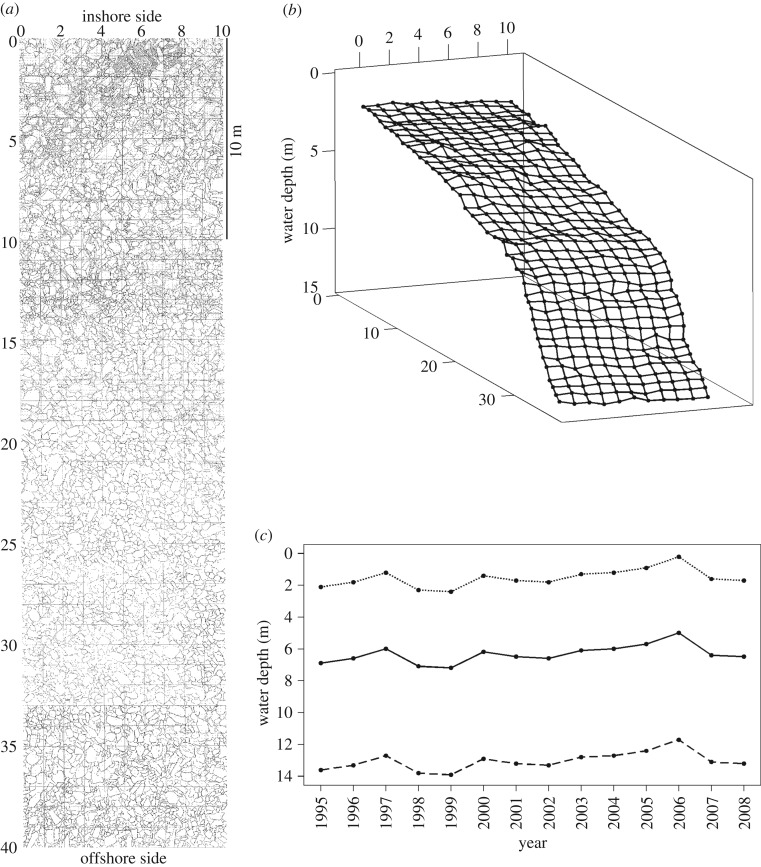
Study quadrat (10 × 40 m) at Kasenga point, southern Lake Tanganyika. (*a*) Substratum map. (*b*) Three-dimensional image of the quadrat. The water depth from 1995 was used. (*c*) Annual changes in water depth from 1995 to 2008. The water depth mean, maximum and minimum are indicated by solid, broken and dotted lines, respectively.

We constructed a 10 × 40 m permanent quadrat and mapped its substratum (figure 1). For 14 years from 1995 to 2008, herbivorous fishes were surveyed every year in the quadrat, and the species composition of every 1 × 1 m^2^ of the quadrat was measured. Then, the census data were analysed to detect the preferences of each species with regard to depth and substratum.

## 2. Material and methods

### 2.1. Field observation of habitat use

A 10 × 40 m quadrat, subdivided with string into 1 × 1 m^2^, was set at depths of 2.1–13.6 m on Kasenga point (8°43′ S, 31°08′ E) near Mpulungu, Zambia, on the southern tip of Lake Tanganyika (figure 1, [5]). All rocks and stones larger than 10 cm on their major axis were mapped, and substrata of gravel and sand areas were also recorded. The substratum within the squares was classified into five types according to the most abundant substrate type: rock (rock size > 50 cm) dominated, stone (20–50 cm) dominated, rubble (3–20 cm) dominated, gravel (0.5–3 cm) dominated and sand (less than 0.5 cm) dominated, following Hori *et al*. [47]. Then, *x-*, *y*- and *z*-values of each node of all the squares in the three-dimensional space were calculated. First, *z*-values were provided by the depth, and *x-* and *y*-values were calculated using the distances from the nearest two nodes using R package nleqslv 3.0 [48] and were plotted in figure 1*b* using the package rgl 0.95 [49]. To estimate the substratum inclination of each square, at first, four triangles were constructed using selected three of four nodes in each square, the normal vector of each triangle was established and the angle between the normal vector and *z*-axis was calculated. Then, substratum inclination of each grid was estimated by averaging four angles between normal vectors and *z*-axis. Note that the substratum inclination indicates the inclination of the 1 × 1 m^2^. These calculations were conducted by R v. 3.2.3 [50].

Fishes in the quadrat were censused by three experts using SCUBA diving in September, October or November once a year from 1995 to 2008. Each census was conducted within 2 or 3 days, spending 6–8 h a day. All fishes used for this study were identified into species during the census. Note that *Petrochromis* sp. (rotundus) in Takeuchi *et al*. [5] has been recognized since 1996 and described as *Petrochromis horii* in 2014 [21]. To avoid counting the same individual redundantly in different squares, the census was conducted from deep side to shallow side of the quadrat, and counted individuals in other squares were carefully removed when observing each square in every census. Each fish individual was classified into a small, medium or large body-size class, probably corresponding to juvenile, sexually immature sub-adult and sexually mature adult, respectively. In this study, only data from adult herbivorous fishes were extracted, and their densities were analysed (see the electronic supplementary material).

### 2.2. Statistical analyses

We analysed the difference of density among fish species within each ecomorph (grazer and browser) in various depth, substratum types and substratum inclination. The number of individuals of each species was analysed by a generalized linear mixed model (GLMM) with depth, substratum type (rock, stone, rubble, gravel or sand dominated), substratum inclination, as fixed factors with the year as a random factor using the R package glmmML 1.0 [51]. For a grazer, *P. horii*, the GLMM was not conducted because of the low frequency of occurrence in the quadrat. Depth and substratum inclination preferences by browsers and grazers were analysed using one-way analysis of variance (ANOVA), and multiple comparisons were analysed using Tukey's test. Variations in depth distribution among substratum types and relationships between depth and substratum inclination were analysed by a generalized liner model (GLM). Tukey's *post hoc* test was applied to compare mean differences in depth between substratum types. To examine whether distance between species in habitat depth was related to phylogenetic relatedness within grazers and browsers in the tribe Tropheini, we compared the phylogenetic distance between each pair of species to their habitat depth difference using the Mantel test with 9999 permutations using the mantel function in vegan 2.2-0 for R [52]. Mitochondrial ND2 and nuclear *ednrb1* and *phpt1* sequences provided by Muschick *et al*. [4] were downloaded from GenBank and aligned with MAFFT 6 ([53]; see the electronic supplementary material for the alignments). Using the combined dataset of the three loci, maximum-likelihood (ML) tree was constructed based on GTR + G model by RAxML 7.7.1 [54]. Then, an ultrametric tree was constructed based on the ML tree using non-parametric rate smoothing by r8s 1.70 ([55]; see the electronic supplementary material, figure S1 for the ultrametric tree). The phylogenetic distance was calculated using the cophenetic function in R. All statistical analyses were conducted using R v. 3.2.3.

## 3. Results

### 3.1. Substratum and depth use in herbivorous cichlids

**Figure 2.**
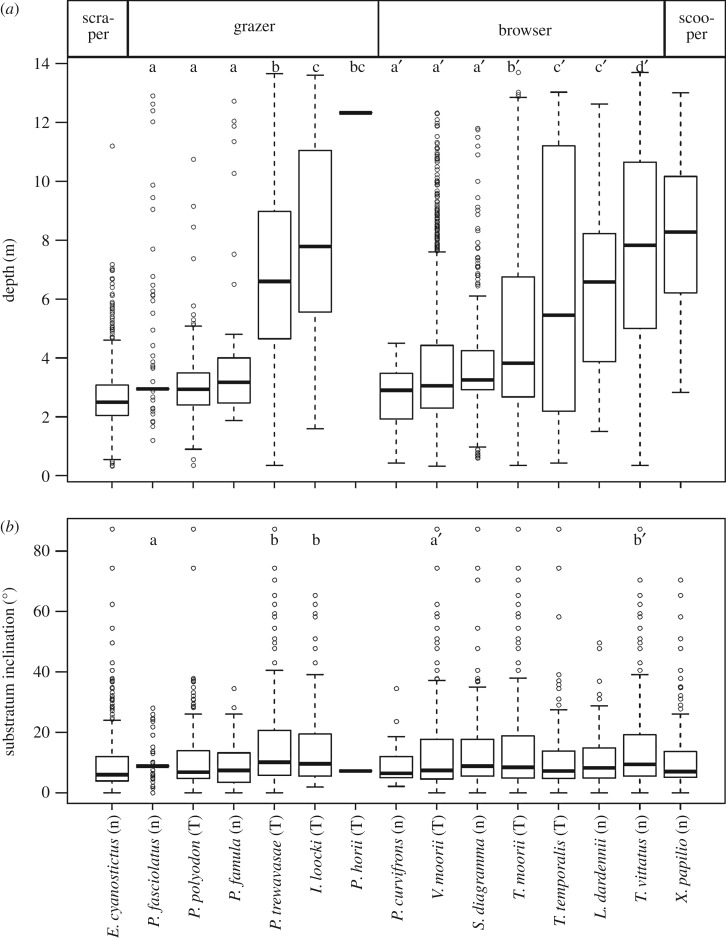
Habitat preferences of herbivorous cichlid species at water depth (*a*) and substratum inclination (*b*) observed from 1995 to 2008 at Kasenga point, southern Lake Tanganyika. Letters following species names indicate cichlid species that defend their feeding territory (T) and species that do not defend any feeding territory (n). Different letters (a–d or a′–d′) on the boxes indicate significant differences among species within grazer and browser, respectively, at 5% by one-way ANOVA followed by Tukey's test.

**Figure 3.**
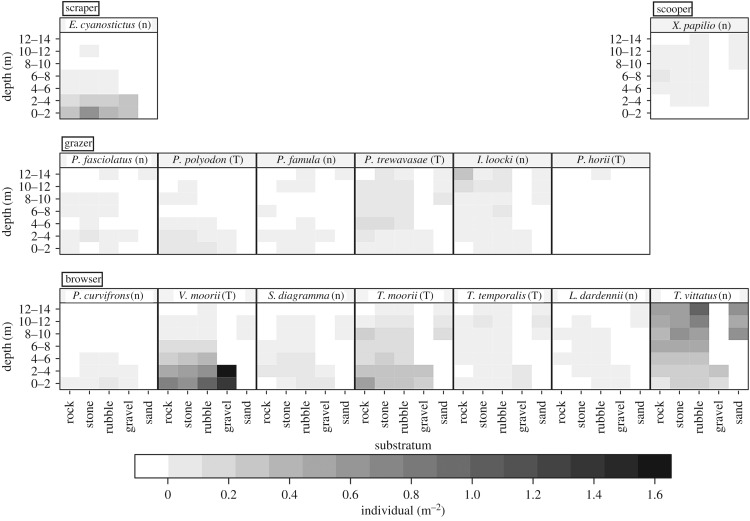
Density of herbivorous cichlids in each depth range and substratum type observed from 1995 to 2008 at Kasenga point, southern Lake Tanganyika. Letters following species names indicate cichlid species that defend their feeding territory (T) and species that do not defend any feeding territory (n).

Grazers and browsers were the richest in species number at this site and comprised six and seven species, respectively. Grazers and browsers used species-specific depth ranges within each ecomorph (figure 2). *Petrochromis fasciolatus*, which had no feeding territory, occurred most frequently in the shallowest zone, overlapping its habitat with a territorial grazer, *Petrochromis polyodon*, and a partially territorial grazer, *Petrochromis famula*. The territorial grazers *Petrochromis trewavasae*, *Interochromis loocki* and *P. horii* inhabited successively deeper zones. Regarding browsers, non-territorial *Pseudosimochromis curvifrons* occupied the shallowest zone, overlapping its depth with territorial *Variabilichromis moorii* and non-territorial *Simochromis diagramma*. Territorial *Tropheus moorii* and *Telmatochromis temporalis*, and non-territorial *Limnotilapia dardennii* and *Telmatochromis vittatus* occurred at successively lower depths. In particular, cichlid species with feeding territories significantly differentiated their habitat depth zones, both within grazers and browsers. Species living in similar depth ranges used separate substratum types within each ecomorph (figures 2*b* and 3). For example, *P. fasciolatus* significantly preferred flat stony substrata compared to the other grazing cichlids, *P. polyodon* and *P. famula*, living in the same shallow area (GLMM, electronic supplementary material, table S1). For browsers, *T. temporalis* preferred gravel in contrast to *L. dardennii*, both inhabiting the intermediate depth zone (electronic supplementary material, table S2). On the other hand, a scraper, *Eretmodus cyanostictus*, inhabited a wide-range of substrata except for sandy bottom in the shallowest zone, and a scooper, *Xenotilapia papilio*, inhabited the relatively deep zone at a low density. Note that the depth distribution differed somewhat among substratum types, that is, gravel- or sand-dominated substrata were located only in the shallow or deep areas, respectively, but rock-, stone- and rubble-dominated substrata were scattered over a wide depth range in our study quadrat (electronic supplementary material, figure S2). On the other hand, there was no significant relationship between depth and substratum inclination (electronic supplementary material, figure S3).

### 3.2. Depth differentiation and phylogenetic distance

**Figure 4.**
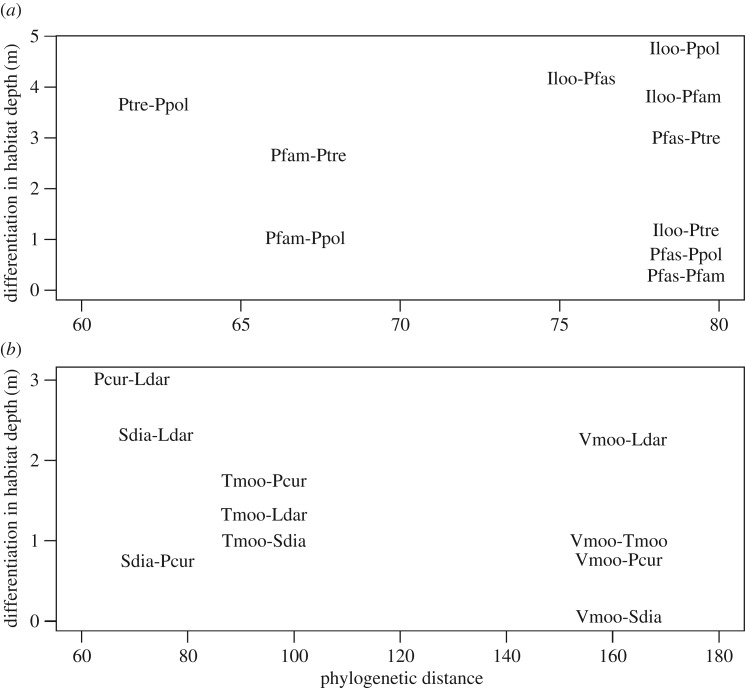
Relationship between the phylogenetic distances of fishes and differentiation in habitat depths of grazers (*a*) and browsers (*b*). See the electronic supplementary material, figure S1 for the scale of phylogenetic distance.

No significant relationship was detected between depth differentiation and phylogenetic distance among grazers, which were composed only of species of the tribe Tropheini (*r* = −0.08, not significant in Mantel test; figure 4), among all browsers (*r* = –0.46, not significant), or among browsers of the tribe Tropheini (*r* = −0.55, not significant).

## 4. Discussion

### 4.1. Depth and substratum differentiations among herbivorous cichlids of the same ecomorph

As a result of our 14-year observation, distinct depth differentiation was detected among grazer species with feeding territories, as well as among browsers with feeding territories. Herbivorous cichlids without feeding territories also exhibited specificity in depth range and substratum use. Carbon stable isotope analyses also confirm that cichlid species depend on primary production produced in their habitat depth [7]. The water level at this site fluctuated within 2.2 m during our 14-year observation (figure 1*c*). The depth range of our study quadrat was 11.5 m, covering most of the depth that herbivorous cichlids inhabit. Considering all the data together, the depth differentiations among cichlid species on a few-metre scale were stable for a long time period in this system, even with the slightly fluctuating water level. The depth and substratum preferences of each cichlid species mostly coincide with observations in Myako, United Republic of Tanzania, on the central east coast of Lake Tanganyika [56], observations in Luhanga, Democratic Republic of the Congo, on the northwestern part of the lake [47], and observations in Kasakalawe and Kalambo, Republic of Zambia, on the southern lake shore [57], although these observations were conducted once to three times in 1 year. In Kasakalawe and Kalambo, *V. moorii* were dominant in gravel- and rubble-dominated substrata in the 0.57–3.57 m depth, and territorial *Petrochromis* spp. were scarce in these habitats. Our observations agree with these findings. In Kasenga, however, *P. fasciolatus* preferred stony substrata, whereas the fish showed no preference for any substrata in Luhanga. This discrepancy may be due to local adaptations of the fish to different environments where substratum availabilities in each depth zone vary and/or to different community structures in component species and their densities. Regarding to the substratum inclination, no significant preference was detected in this study. One potential reason for the lack of significant preference detected is that we measured inclination for each 1 × 1 m^2^. In each square, substratum is composed of stones, rubbles, gravels and/or sands to make the inclination heterogeneous in a smaller scale, and cichlids possibly chose specific inclination of substrata for feeding and nesting within the 1 × 1 m^2^.

### 4.2. Overlap of habitat between species of different ecomorphs

Both grazers and browsers separate their home ranges among species by depth and substratum type, but many pairs of grazers and browsers share the same habitats (figures 2 and 3). It is known that species of different ecomorphs segregate their niches sufficiently to avoid competing with each other and can coexist sympatrically [58–60]. In Tanganyikan herbivorous cichlids, different ecomorphs segregate their diets by using different feeding strategies [6,12,27,28], and the coexistence of species of different ecomorphs was also observed in this study on rocky substrata, especially in the shallowest zone where light intensity and the rate of photosynthesis are highest in the lake [61,62]. In particular, the grazer *P. polyodon* and the browser *T. moorii* were frequently observed to overlap their feeding territories, and they are described to have mutualistic interactions in which territorial defence by one species contributes to the territoriality of the other, and grazing by *P. polyodon* seems to remove sand together with epiphyton from mats of filamentous algae/cyanobacteria, making browsing on the algae easier for *T. moorii* [47,63].

### 4.3. The proximate causes of depth differentiations among herbivorous cichlids of the same ecomorph

A metagenomic approach based on 16S rRNA gene revealed that periphyton assemblages inside territories are determined by habitat depth and therefore, cichlid species inhabiting a specific depth have a specific structure of periphyton [6]. Additionally, cichlid species ingest some cyanobacteria and diatoms selectively and, finally, stomach contents were quite different among species, even among species in the same feeding ecomorph [6]. This means that the variations in algal/cyanobacterial communities with depth might be a proximate cause of specialization by herbivorous cichlids in a specific depth zone observed in this study. The depth variations in algal/cyanobacterial communities may be caused by variations in light intensity and light transmission and varying frequencies of disturbances at different depths [64,65].

The differences in light transmission with depth may also be a more directly proximate cause of depth segregation among cichlids, as observed in Lake Victoria [46,66,67]. Cichlids in the lake have undergone divergent selection of male nuptial colours at different depths in which light environments vary, corresponding with female opsin genes that affect colour vision, and have parapatrically diversified, resulting in some pairs of sister species inhabiting neighbouring depths. In Tanganyikan cichlids, functional divergence in the rhodopsin gene and *short wavelength-sensitive 2b* gene was also observed between cichlids in deep water and those in shallow water [68–70]. Further study may reveal a more detailed functional diversification of sight in Tanganyikan herbivorous cichlids adapted to habitat depth within the shallow area observed in this study.

Severe competition for space among species in the same ecomorph also enhances depth differentiation among species [12,27,41]. In the central eastern section of Lake Tanganyika, *Tropheus polli* coexists with *T. moorii* ‘Kirschfleck’ at Mabilibili point and with *T. moorii* ‘Kaiser’ at Kekese point. At these sites, they live separately in neighbouring depths, but at Ikola point *T. moorii* ‘Kaiser’ lives alone and occupies the entire depth range [71]. Depth differentiation by sympatric *Tropheus* species and consequent morphological differentiations adapting to each habitat depth seem to be triggered by competition.

Substratum preferences of cichlids can also be a proximate factor of depth differentiation. In general, gravel and boulder substrata near the shoreline are sorted by size and weight along elevation, controlled by beach slope, wave power and supply of gravel/boulders from land [72–74]. Beach slope receives feedback and is determined by accumulation and removal of substrata. In this way, the substratum is correlated with water depth. In our study site, the substratum seems to be partially sorted (figure 1*a*), although rock-, stone- and rubble-dominated substrata were evenly distributed throughout the depth range. To distinguish between the depth and substratum preferences of these cichlids, comparisons of depth/substratum utilization are necessary among various sites where beach slope, supply of boulders and wave power vary.

Regarding the different risks of predation at various depths, nine species of piscivores and two species of scale eaters were observed at our study site [5]. The densities of these fishes were almost equal for all substrata and all depth ranges, and therefore, they are unlikely to affect the depth and substratum utilization of herbivorous fishes [5], although piscivores can alter the distribution of herbivores at smaller scales, as potential prey cichlids maintain a 10 cm distance from the piscivore *Lamprologus elongatus* [47]. On the other hand, bird predators are suggested to be more influential in the shallow area than in the deep area [71], but further observation is needed.

### 4.4. Possible evolutionary process of acquiring depth differentiation and its consequences

Most of these herbivorous cichlids (e.g. all species of Tropheini, Ectodini, Eretmodini) are mouth-brooders, and mouth-brooding enables the reproduction of these fish to be independent of the substratum in shallow water where waves cause intense disturbances [56]. This may lead to specialization of these herbivorous cichlids in shallower areas and to depth differentiation on a finer scale. Under stenotopy for specific depths and substrata, each population undergoes local selection, and gene flow between populations living in different environments can be restricted. Furthermore, when habitats are separated, ancestral species can diversify into different environments and specialize enough so as not to be mixed with each other after habitats are reunified and these populations re-encounter each other. In Lake Victoria, cichlid species specialize in different depth ranges and have diversified within several metres of depth [46]. Furthermore, repeated lake-level fluctuation is thought to drive diversification of Tanganyikan cichlids through the repetitive shrinking and expansion of habitats [75]. A Tanganyikan cichlid, *T. temporalis*, has diversified into two genetically distinct ecomorphs: a large-bodied rock-living ecomorph and a small-bodied shell-living ecomorph [76,77]. This diversification occurred repeatedly in places where rocky habitats and shell beds were neighbouring. Therefore, a variant that was small in size when mature, originating from the original population in the rocky habitat, is thought to have shifted to the shell bed when the shell bed reached a suitable depth due to lake-level changes [77].

Our 14-year observations on habitat depth and substratum preferences of herbivorous cichlids on a rocky littoral slope in Lake Tanganyika revealed the clear depth differentiation among grazers and browsers with feeding territories. Additionally, herbivorous cichlids without feeding territories also exhibited specificity in depth range and substratum use, resulting in habitat segregation among species of herbivorous cichlids belonging to the same feeding ecomorphs. This niche segregation may have been acquired by competition between equivalent species encountered through repetitive lake-level fluctuation.

## Supplementary Material

Additional file 1. Raw data of census survey of herbivorous cichlids at Kasenga Point in Lake Tanganyika in 1995–2006

## Supplementary Material

Additional file 2. Aligned mitocondrial ND2 sequences of Tanganyikan cichlids cited from Muschick et al. [4].

## Supplementary Material

Additional file 3. Aligned nuclear ednrb1 sequences of Tanganyikan cichlids cited from Muschick et al. [4].

## Supplementary Material

Additional file 4. Aligned nuclear phpt1 sequences of Tanganyikan cichlids cited from Muschick et al. [4].

## Supplementary Material

Table S1. Generalised linear mixed model of the density of grazing herbivorous cichlids. Cichlid species, depth, substratum type, and inclination of substratum were analysed as fixed factors with survey year as a random factor. Std. Error = standard error.

## Supplementary Material

Table S2. Generalised linear mixed model of the density of browsing herbivorous cichlids. Cichlid species, depth, substratum type, and inclination of substratum were analysed as fixed factors with survey year as a random factor. Std. Error = standard error.

## Supplementary Material

Figure S1. Maximum likelihoodUltrametric tree of Tanganyikan cichlids based on mitochondrial ND2 and nuclear ednrb1 and phpt1 sequences published in the previous study [4].

## Supplementary Material

Figure S2. Box plot of depth variation of substratum types in the observed 10 ˟ 40-m quadrat at Kasenga point, southern Lake Tanganyika. Different letters on the boxes indicate significant differences at 5% by Tukey's test.

## Supplementary Material

Figure S3. Relationships between substratum inclination and depth in the observed 10 ˟ 40-m quadrat at Kasenga point, southern Lake Tanganyika. Different letters on the boxes indicate significant differences at 5% by Tukey's test.

## Data accessibility

The datasets supporting this article have been uploaded as the electronic supplementary material.
