# Supplementary material for: Depth and substratum differentiations among coexisting herbivorous cichlids in Lake Tanganyika
Source: R Soc Open Sci. 2016 Nov 16;3(11):160229. doi: 10.1098/rsos.160229 (PMC5180107; doi:10.1098/rsos.160229)
Supplement: Table S1. Generalised linear mixed model of the density of grazing herbivorous cichlids. Cichlid species, depth, substratum type, and inclination of substratum were analysed as fixed factors with survey year as a random factor. Std. Error = standard error. [file rsos160229supp5.docx]

Table S1. Generalised linear mixed model of the density of grazing herbivorous cichlids. Habitat depth, substratum type, and inclination of substratum were analysed as fixed factors with survey year as a random factor. * indicates significant after Bonferroni correction.

coefficients standard z value *p*

error

*I. loocki*

(Intercept) -3.17 0.34 -9.29 0.0000 *

depth 0.56 0.06 9.55 0.0000 *

substratum (stone) -0.09 0.19 -0.49 0.6220

substratum (rubble) -0.21 0.19 -1.07 0.2830

substratum (gravel) 0.10 0.39 0.25 0.8000

substratum (sand) -0.33 0.26 -1.27 0.2050

inclination 0.05 0.05 0.91 0.3650

*P. famula*

(Intercept) -6.12 0.80 -7.63 0.0000 *

depth -0.94 0.24 -3.91 0.0001 *

substratum (stone) 0.26 0.76 0.34 0.7310

substratum (rubble) 0.58 0.74 0.78 0.4370

substratum (gravel) -0.14 0.93 -0.15 0.8820

substratum (sand) 1.46 1.31 1.11 0.2660

inclination -0.31 0.21 -1.47 0.1420

*P. fasciolatus*

(Intercept) -5.71 0.56 -10.21 0.0000 *

depth -1.39 0.15 -9.36 0.0000 *

substratum (stone) 0.97 0.37 2.67 0.0076 *

substratum (rubble) -0.87 0.40 -2.16 0.0308

substratum (gravel) -0.46 0.46 -1.00 0.3150

substratum (sand) 1.68 0.85 1.97 0.0490

inclination -0.54 0.12 -4.57 0.0000 *

*P. polyodon*

(Intercept) -4.17 0.31 -13.35 0.0000 *

depth -2.13 0.18 -11.81 0.0000 *

substratum (stone) -0.33 0.24 -1.41 0.1600

substratum (rubble) -0.48 0.24 -2.02 0.0431

substratum (gravel) -0.83 0.31 -2.73 0.0064 *

substratum (sand) -27.78 2282000 0.00 1.0000

inclination 0.09 0.06 1.53 0.1260

*P. trewavasae*

(Intercept) -1.71 0.12 -14.55 0.0000 *

depth 0.14 0.04 3.55 0.0004 *

substratum (stone) -0.16 0.12 -1.34 0.1810

substratum (rubble) -0.44 0.13 -3.49 0.0005 *

substratum (gravel) -1.12 0.31 -3.56 0.0004 *

substratum (sand) -1.03 0.25 -4.19 0.0000 *

inclination 0.08 0.03 2.49 0.0128
