# Supplementary material for: Depth and substratum differentiations among coexisting herbivorous cichlids in Lake Tanganyika
Source: R Soc Open Sci. 2016 Nov 16;3(11):160229. doi: 10.1098/rsos.160229 (PMC5180107; doi:10.1098/rsos.160229)
Supplement: Table S2. Generalised linear mixed model of the density of browsing herbivorous cichlids. Cichlid species, depth, substratum type, and inclination of substratum were analysed as fixed factors with survey year as a random factor. Std. Error = standard error. [file rsos160229supp6.docx]

Table S2. Generalised linear mixed model of the density of browsing herbivorous cichlids. Habitat depth, substratum type, and inclination of substratum were analysed as fixed factors with survey year as a random factor. * indicates significant after Bonferroni correction.

coefficients standard z value *p*

error

*L. dardenii* (Intercept) -3.29 0.28 -11.73 0.0000 *

depth -0.06 0.07 -0.88 0.3800

substratum (stone) 0.04 0.24 0.17 0.8660

substratum (rubble) 0.02 0.24 0.06 0.9500

substratum (gravel) 0.33 0.33 1.00 0.3180

substratum (sand) -0.08 0.40 -0.20 0.8390

inclination -0.16 0.07 -2.12 0.0341

*V. moorii* (Intercept) -0.88 0.10 -8.43 0.0000 *

depth -1.10 0.03 -40.56 0.0000 *

substratum (stone) 0.03 0.07 0.44 0.6580

substratum (rubble) 0.16 0.07 2.49 0.0128

substratum (gravel) 0.14 0.08 1.74 0.0821

substratum (sand) -1.17 0.39 -3.02 0.0025 *

inclination 0.03 0.02 1.88 0.0608

*P. curvifrons* (Intercept) -7.78 1.16 -6.71 0.0000 *

depth -2.18 0.50 -4.37 0.0000 *

substratum (stone) 0.65 1.04 0.63 0.5300

substratum (rubble) 0.85 1.03 0.82 0.4100

substratum (gravel) 0.47 1.14 0.41 0.6800

substratum (sand) -14.33 13350 0.00 0.9990

inclination 0.12 0.16 0.78 0.4350

*S. diagramma* (Intercept) -3.35 0.26 -13.10 0.0000 *

depth -1.35 0.08 -16.57 0.0000 *

substratum (stone) 0.38 0.19 1.99 0.0467

substratum (rubble) 0.23 0.19 1.17 0.2420

substratum (gravel) -0.45 0.26 -1.75 0.0794

substratum (sand) 0.28 0.75 0.37 0.7100

inclination 0.03 0.04 0.75 0.4540

*T. moorii* (Intercept) -1.26 0.14 -8.86 0.0000 *

depth -0.65 0.03 -21.34 0.0000 *

substratum (stone) 0.01 0.09 0.12 0.9070

substratum (rubble) 0.03 0.09 0.32 0.7480

substratum (gravel) -0.53 0.13 -4.00 0.0001 *

substratum (sand) -0.09 0.21 -0.41 0.6810

inclination -0.01 0.02 -0.62 0.5360

*T. temporalis* (Intercept) -4.00 0.35 -11.33 0.0000 *

depth 0.36 0.09 3.98 0.0001 *

substratum (stone) 0.01 0.35 0.02 0.9800

substratum (rubble) 0.25 0.34 0.73 0.4680

substratum (gravel) 2.86 0.37 7.65 0.0000 *

substratum (sand) 0.84 0.40 2.13 0.0335

inclination 0.05 0.08 0.68 0.4990

*T. vittatus* (Intercept) -0.41 0.09 -4.60 0.0000 *

depth 0.48 0.02 29.49 0.0000 *

substratum (stone) 0.09 0.06 1.53 0.1260

substratum (rubble) 0.09 0.06 1.57 0.1170

substratum (gravel) 0.05 0.12 0.42 0.6780

substratum (sand) -0.28 0.08 -3.33 0.0009 *

inclination 0.03 0.02 1.67 0.0953
