# Supplementary figures and images for: Depth and substratum differentiations among coexisting herbivorous cichlids in Lake Tanganyika
Source: R Soc Open Sci. 2016 Nov 16;3(11):160229. doi: 10.1098/rsos.160229 (PMC5180107; doi:10.1098/rsos.160229)

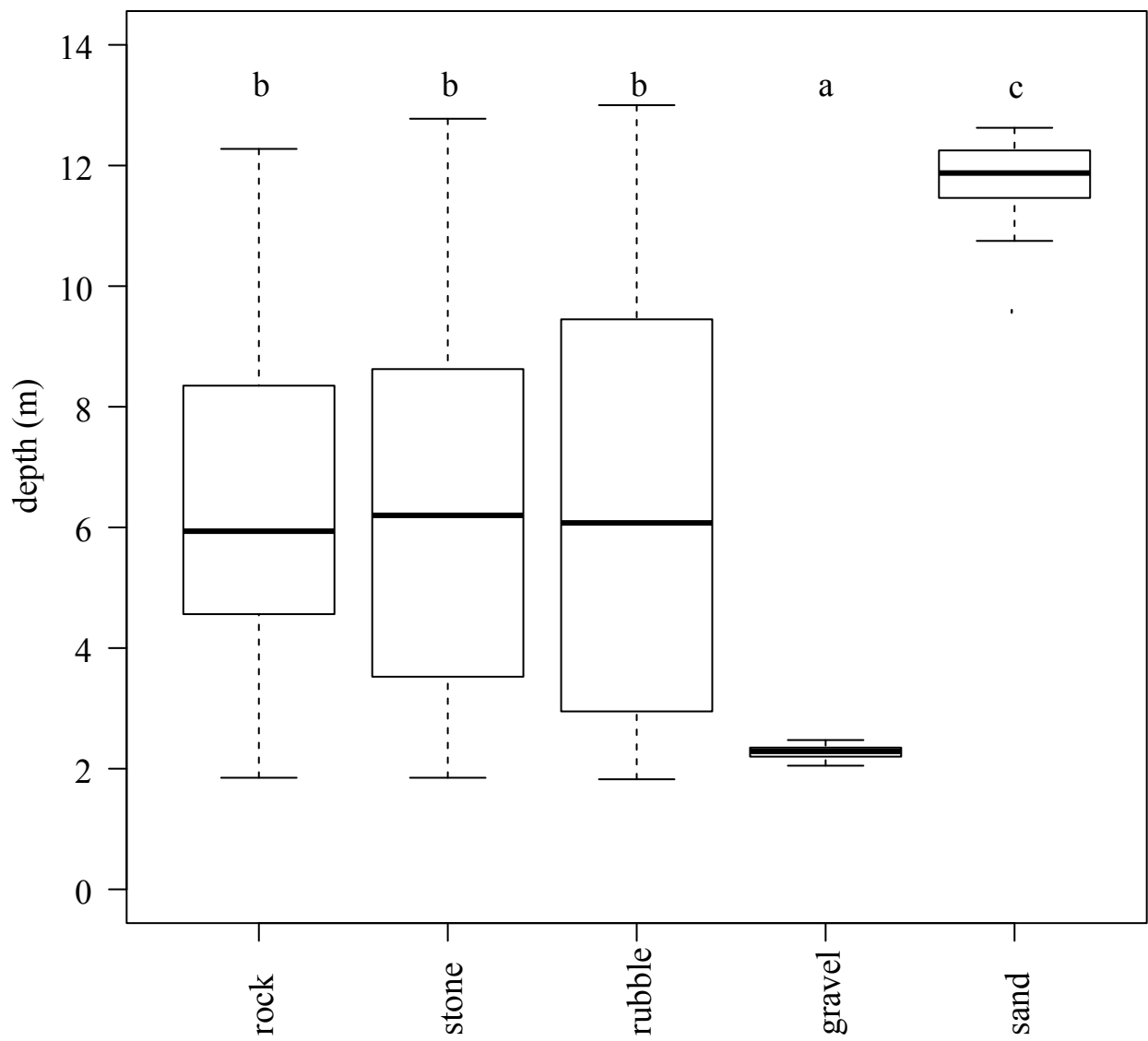

Fig. S2

Supplement: Figure S2. Box plot of depth variation of substratum types in the observed 10 ˟ 40-m quadrat at Kasenga point, southern Lake Tanganyika. Different letters on the boxes indicate significant differences at 5% by Tukey's test. [file rsos160229supp8.pdf]

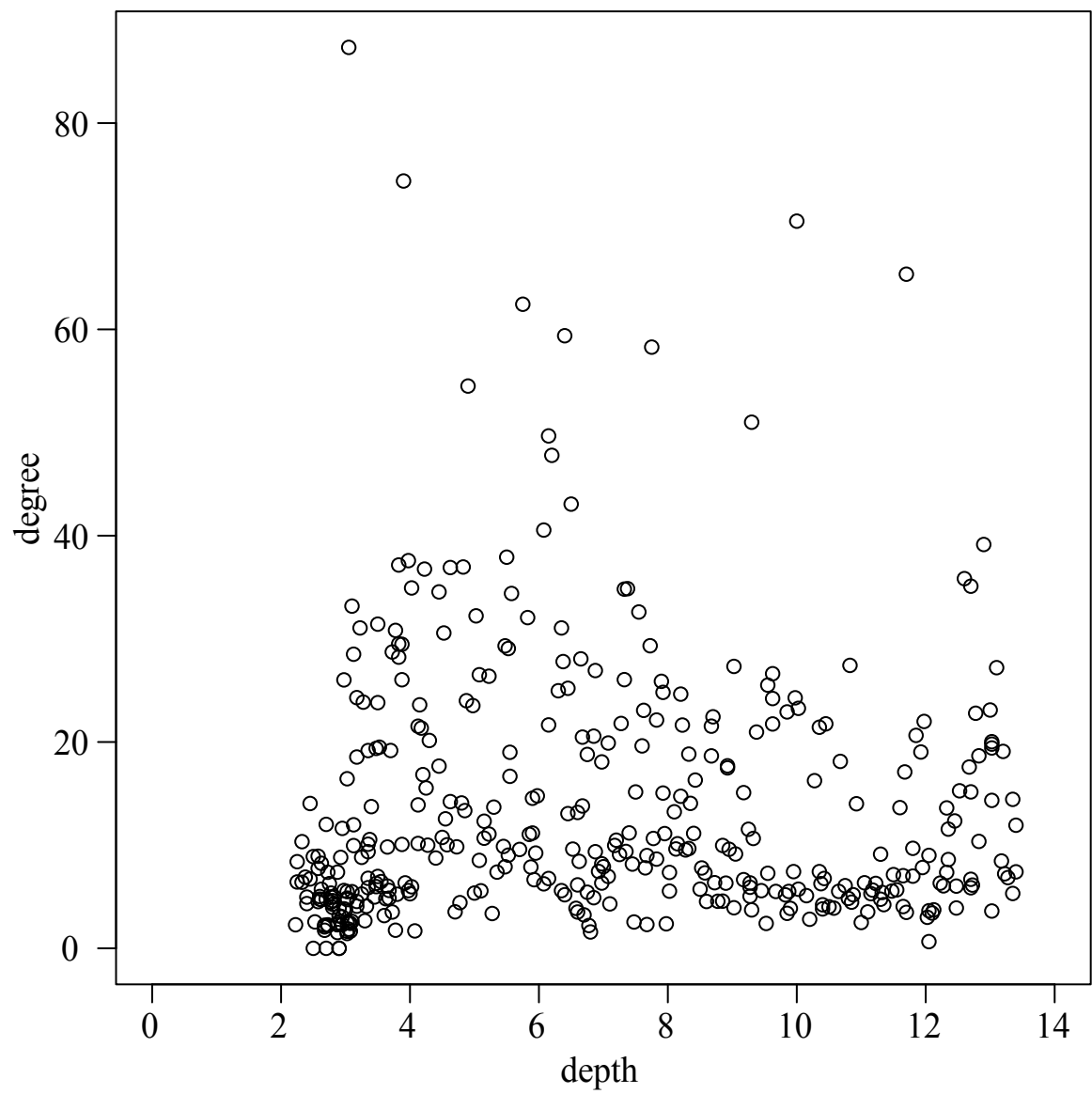

Fig. S3

Supplement: Figure S3. Relationships between substratum inclination and depth in the observed 10 ˟ 40-m quadrat at Kasenga point, southern Lake Tanganyika. Different letters on the boxes indicate significant differences at 5% by Tukey's test. [file rsos160229supp9.pdf]
